# Efficacy of cyclin-dependent kinase inhibitors with concurrent proton pump inhibitors in patients with breast cancer: a systematic review and meta-analysis

**DOI:** 10.1093/oncolo/oyae320

**Published:** 2025-02-18

**Authors:** Zijie Guo, Ziyu Zhu, Mingpeng Luo, Yijia Cao, Xixi Lin, Qingliang Wu, Shenkangle Wang, Linbo Wang, Jichun Zhou

**Affiliations:** Department of Surgical Oncology, Affiliated Sir Run Run Shaw Hospital, Zhejiang University School of Medicine, Hangzhou, Zhejiang 310016, People’s Republic of China; Biomedical Research Center and Key Laboratory of Biotherapy of Zhejiang Province, Hangzhou, Zhejiang 310016, People’s Republic of China; Department of Surgical Oncology, Affiliated Sir Run Run Shaw Hospital, Zhejiang University School of Medicine, Hangzhou, Zhejiang 310016, People’s Republic of China; Biomedical Research Center and Key Laboratory of Biotherapy of Zhejiang Province, Hangzhou, Zhejiang 310016, People’s Republic of China; Department of Surgical Oncology, Affiliated Sir Run Run Shaw Hospital, Zhejiang University School of Medicine, Hangzhou, Zhejiang 310016, People’s Republic of China; Biomedical Research Center and Key Laboratory of Biotherapy of Zhejiang Province, Hangzhou, Zhejiang 310016, People’s Republic of China; The First Affiliated Hospital of Zhejiang Chinese Medical University, Hangzhou, Zhejiang 310014, People’s Republic of China; Department of Surgical Oncology, Affiliated Sir Run Run Shaw Hospital, Zhejiang University School of Medicine, Hangzhou, Zhejiang 310016, People’s Republic of China; Department of Surgical Oncology, Affiliated Sir Run Run Shaw Hospital, Zhejiang University School of Medicine, Hangzhou, Zhejiang 310016, People’s Republic of China; Biomedical Research Center and Key Laboratory of Biotherapy of Zhejiang Province, Hangzhou, Zhejiang 310016, People’s Republic of China; Department of Surgical Oncology, Affiliated Sir Run Run Shaw Hospital, Zhejiang University School of Medicine, Hangzhou, Zhejiang 310016, People’s Republic of China; Biomedical Research Center and Key Laboratory of Biotherapy of Zhejiang Province, Hangzhou, Zhejiang 310016, People’s Republic of China; The Ninth People’s Hospital of Hangzhou, Hangzhou, Zhejiang 310014, People’s Republic of China; Department of Surgical Oncology, Affiliated Sir Run Run Shaw Hospital, Zhejiang University School of Medicine, Hangzhou, Zhejiang 310016, People’s Republic of China; Biomedical Research Center and Key Laboratory of Biotherapy of Zhejiang Province, Hangzhou, Zhejiang 310016, People’s Republic of China; Department of Surgical Oncology, Affiliated Sir Run Run Shaw Hospital, Zhejiang University School of Medicine, Hangzhou, Zhejiang 310016, People’s Republic of China; Biomedical Research Center and Key Laboratory of Biotherapy of Zhejiang Province, Hangzhou, Zhejiang 310016, People’s Republic of China; Department of Surgical Oncology, Affiliated Sir Run Run Shaw Hospital, Zhejiang University School of Medicine, Hangzhou, Zhejiang 310016, People’s Republic of China; Biomedical Research Center and Key Laboratory of Biotherapy of Zhejiang Province, Hangzhou, Zhejiang 310016, People’s Republic of China

**Keywords:** cyclin-dependent kinase inhibitor, proton pump inhibitor, breast cancer, survival rate, drug-related side effects and adverse reactions, systematic review

## Abstract

**Background:**

The impact of concurrent proton pump inhibitors (PPIs) use on the prognosis of patients with breast cancer undergoing cyclin-dependent kinase inhibitors (CDKIs) treatment is currently uncertain. Considerable divergence exists regarding the clinical studies. In this study, we aim to perform a comprehensive analysis to evaluate the influence of concomitant PPI use on the effectiveness and adverse effects of CDKIs in patients with breast cancer.

**Methods:**

This study encompassed all pertinent clinical studies published up to the present, following the PRISMA guidelines. The study used hazard ratio (HR) or odds ratio (OR) as a summary statistic and used fixed or random effects models for pooled estimation.

**Results:**

This study incorporated 10 research articles involving 2993 participants. Among patients with breast cancer undergoing treatment with CDKIs, the simultaneous administration of PPIs was associated with a notable reduction in overall survival (HR = 2.00; 95% CI, 1.35-2.96). Nevertheless, no substantial correlation was observed between the simultaneous utilization of PPIs and the progression-free survival (PFS) of patients (HR = 1.30; 95% CI, 0.98-1.74). PFS did not change significantly when considering different drugs, treatment lines, or regions alone. Furthermore, the simultaneous administration of PPIs was found to result in a notable decrease in the incidence of grades 3/4 risk factors (OR = 0.63, 95% CI, 0.46-0.85).

**Conclusion:**

The concurrent administration of PPIs did not result in significant alterations in the risk of disease advancement among patients with breast cancer undergoing CDKIs treatment. The utilization of PPIs led to a decrease in the adverse effects linked to the administration of CDKIs.

**Implications for practice**

The concurrent administration of CDKIs and PPIs in the treatment of breast cancer patients has prompted inquiries into its potential impact on treatment efficacy. This study is the first comprehensive meta-analysis aimed at examining the effectiveness, adverse effects, and subgroup efficacy of concomitant employment of PPIs with CDKIs in patients with breast cancer. Through analysis, the fundamental safety and advantages of employing PPIs in the therapy of patients with breast cancer utilizing CDKIs have been ascertained. This finding will reduce apprehensions for both patients and doctors when devising treatment strategies, thereby enhancing the survival rates and quality of patients with breast cancer.

## Introduction

In recent years, there has been a notable rise in the prevalence of breast cancer, making it the most common form of cancer among women in numerous countries and regions.^1^ The management and outlook of breast cancer have emerged as significant global concerns. Enhancing the treatment of patients with breast cancer and improving their prognosis and quality of life are major priorities for oncologists and breast surgeons. According to the prevailing consensus, breast cancers are classified into 3 primary types depending on surface receptor expression, with the hormone receptor (HR) positive/human epidermal growth factor 2 (HER2) negative subtype being the most prevalent, encompassing over 70% of patients.^2,3^ Recently, CDKIs, including palbociclib, ribociclib, and abemaciclib, have been established as the standard treatment for patients with HR+/HER2− breast cancer.^4^ They can be used not only in initial or secondary treatment protocols for individuals with advanced metastatic breast cancer but also as supplementary therapy for patients with high-risk, early-stage breast cancer.^4,5^ Several clinical studies have shown a notable enhancement in the prognosis of individuals with advanced breast cancer through the combination of CDKIs with endocrine medications like letrozole and fulvestrant.^6-8^

PPIs are widely acknowledged as the gold standard drugs for managing gastrointestinal symptoms in patients with cancer.^9^ Some studies have indicated that PPIs could potentially impact the microecological balance within the gastrointestinal tract and intragastric pH levels. Consequently, this alteration may influence the effectiveness of certain anticancer medications,^10,11^ especially orally administered anticancer medications.^12^ CDKIs are commonly prescribed in the form of oral tablets or capsules. Moreover, considering the pharmacokinetic aspect, CDKIs are characterized as alkaline compounds and optimal absorption is favored in a stable and highly acidic gastric pH environment.^13^ Consequently, the inquiry into the potential impact of PPIs on the effectiveness of CDKIs has emerged as a significant concern among researchers and healthcare professionals.

From a clinical practice perspective, numerous clinical studies have been conducted to directly examine the prognostic implications, encompassing efficacy, or to say survival outcomes, and side effects, of PPIs in patients with breast cancer undergoing CDKIs treatment. Several studies have indicated the potential implications of PPIs on the effectiveness and side effects of CDKIs. While some studies propose that the concurrent use of these medications does not impact the outcome for patients with breast cancer, others suggest a correlation between prognosis and variables such as the specific type of medication administered. A systematic review of Chang et al demonstrated that the survival outcomes of patients with breast cancer receiving palbociclib were significantly and adversely impacted when concomitantly treated with PPIs, while ribociclib did not have a similar effect.^14^ However, a systematic review conducted by de Moraes et al determined that the survival outcomes of patients with breast cancer receiving treatment with either palbociclib or ribociclib were notably and adversely affected when administered concurrently with PPIs.^15^ These studies demonstrated innovation and advancement, yet it also prompted numerous inquiries for clinicians arising from their clinical practice and the patients’ concerns. Besides, these reviews do not reach a consensus view. Following the in-depth exploration of methodologies for systematic reviews of relevant topics and the recent publication of several new clinical studies,^16,17^ our researchers posit that a reassessment of the relevant conclusion may be justified. Furthermore, it is imperative to investigate the prognostic implications of simultaneous PPIs and CDKIs usage in patients with breast cancer comprehensively, considering the potential-related complications. Furthermore, it is imperative to conduct several crucial subgroup analyses on distinct patient cohorts.

Given the rising number of patients with breast cancer undergoing treatment with CDKIs and the potential implications of combining PPIs with CDKIs for these patients, it is imperative that new systematic assessments and meta-analyses be conducted promptly. Therefore, the researchers conducted a thorough review of the pertinent literature, and carried out a new systematic review and meta-analysis with more extensive and precise subgroup analyses. The aim was to enhance the understanding of the correlation between the simultaneous use of PPIs and the effectiveness and adverse effects of CDKI therapy in patients with breast cancer and offer a clinical treatment approach for the integration of patients with breast cancer.

## Materials and methods

The study adhered to the guidelines of PRISMA.^18,19^ This study was prospectively registered on PROSPERO on March 28, 2024, with the registration number CRD42024529742.

### Search strategy

A thorough electronic search was carried out across multiple databases including PubMed, Web of Science, Embase, Cochrane Library, and ClinicalTrials.gov. The search will cover the period from the establishment of the database to April 30, 2024. After cross-referencing with the PubMed Medical Subjects Headings (MeSH), the search terms used include “blast cancer,” “CDK inhibitor” or “CDKI” or “Palbociclib” or “Ribociclib” or “Abemaciclib,” “proton pump inhibitors” or “PPI” or “gastric acid suppressant” or “H2 blocker.” All the retrieved articles were manually downloaded in full text from relevant publication websites. Subsequently, they were consolidated into a database using *Endnote X9* to eliminate duplicates and ensure consistent management. The most recent search was conducted on May 5, 2024.

### Inclusion criteria and study eligibility

The study population consisted of patients with breast cancer who received PPIs in conjunction with CDKI therapy, while the control group comprised patients with breast cancer who did not receive PPIs alongside CDKI therapy. The term “Concomitant use of PPIs” was operationally defined as individuals being exposed to PPIs for a minimum of one-third of the duration of CDKI therapy and commencing PPI treatment no later than the initiation of CDKI therapy. The study endpoints encompassed at least one of the survival outcomes documented in the literature for individuals diagnosed with breast cancer, such as overall survival (OS) and progression-free survival (PFS). All articles included in this analysis had to meet specific inclusion criteria. These criteria encompassed the availability of the complete full text, articles written in English, inclusion of complete keywords, and provision of data on at least one survival outcome. In terms of article selection, this study encompassed case-control studies, retrospective studies, prospective cohort studies, and randomized controlled trials.

Following the initial screening based on the criteria, 2 independent reviewers (Z.G. and Z.Z.) meticulously examined the selected studies to exclude any literature that did not align with the study’s objectives. In cases of discrepancies, discussions were held with the co-investigators to address and achieve a consensus.

### Data extraction

After 2 independent investigators conducted the article screening based on the predetermined inclusion criteria and reached a consensus on the literature selected for subsequent analysis, the data extracted from the articles included general information (eg, “author,” “publication year,” “region,” “study period”), study characteristics (eg, “population,” “sample size”), participant characteristics (eg, “adjustment factors,” “CDKIs treatment regimen,” “time and window of PPIs administration,” “ECOG-PS”), as well as “survival outcomes” and “side effects.” Survival outcomes included OS and PFS. In cases where data were missing, efforts were made to contact the corresponding author for additional information. All communication regarding missing data is thoroughly documented in the supplementary material. To minimize the impact of adjusting factors on the results, adjusted hazard ratios (HRs) reported in the articles were directly extracted. In instances where HRs were not directly provided in the study or in the requested data but were only represented by Kaplan-Meier survival curves, *Engauge Digitizer,* and related computational methods were used to estimate HRs from the curves.^20^ For pooled odds ratios (ORs) related to side effect profiles, direct values were used when available; otherwise, calculations were based on the provided data.

### Quality assessment and control of bias

Several diverse methodologies were used to assess the quality of the articles. Two investigators (Z.G. and Z.Z.) autonomously evaluated the risk of bias by employing the ROB 2.0^21^ and the Standardized Robins-I Tool.^22^ Additionally, they assessed the quality of the studies using the Newcastle-Ottawa Scale (NOS).^23^ Publication bias was evaluated through visual examination of forest plots by 2 researchers. Disagreements were resolved through consultations between the 2 investigators and the joint investigators to achieve consensus.

### Statistical analysis

All analyses in this study were conducted utilizing *Review Manager 5.3* with a predetermined statistical significance level of *P* < .05. Forest plots were used for assessing the risk of breast cancer incidence. For continuous variables, we used the weighted mean difference as a measure to quantify variance. HRs for survival outcomes will be summarized utilizing the inverse variance method. For the hazard ratios related to side effect outcomes, we aggregated the hazard ratios from individual studies utilizing the Mantel-Haenszel method, with OR serving as the pooled risk scale value.

The assessment of heterogeneity among the studies will be conducted through the Cochrane’s *Q* test and *I*² statistic.^24^ When substantial heterogeneity (*i* ≥ 50% in the Cochrane *Q* test) is detected, the random effects (DerSimonian-Laird) model will be used for the overall estimate; alternatively, the fixed effects model will be used for the comprehensive estimate.^25^ A significance level of *P* < .050 was deemed statistically significant.

## Results

### Study selection

**Figure 1.**
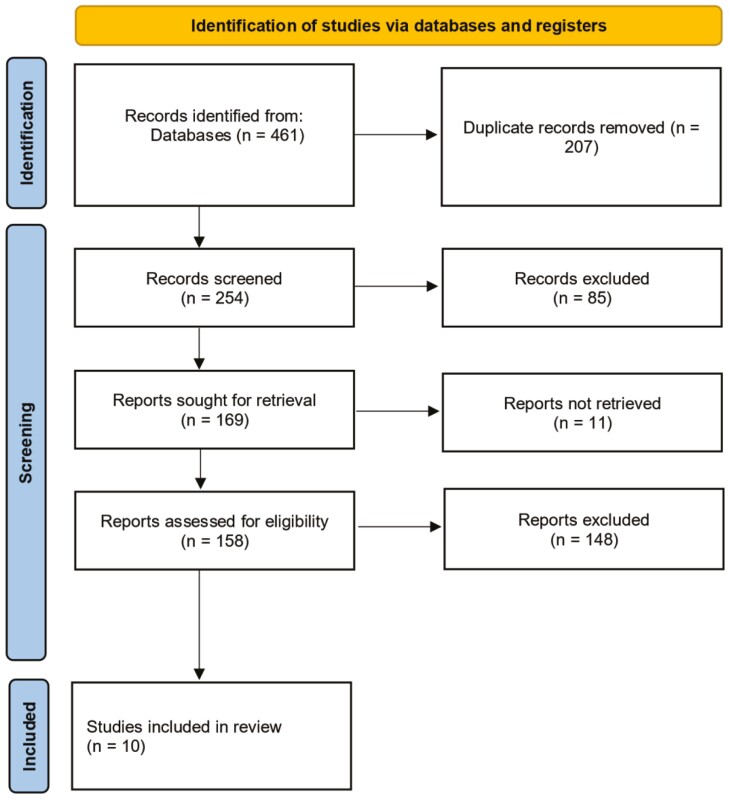
PRISMA flow diagram of the study selection.

A total of 461 articles were screened across 5 databases for inclusion in the primary study and subsequent review. After eliminating duplicates from various databases (*n* = 207), the remaining 254 research articles were subsequently evaluated. Upon a rapid assessment of the articles, 96 were excluded for not meeting the inclusion criteria. Subsequently, a more detailed examination was conducted on the titles, abstracts, and keywords of the remaining 158 articles, leading to the elimination of 148 articles that did not align with the objectives of this review. Ten articles that met the objectives and inclusion criteria for this review and analysis of clinical studies were ultimately selected for further review and analysis. The flow of the included articles is depicted in Figure 1.

### Characteristics of the included studies

Among the 10 studies encompassed in this systematic review,^13,16,17,26-32^ a collective total of 2993 participants were involved. It is noteworthy that certain articles did not furnish pertinent data explicitly, leading to speculation on relevant databased on the content of the article. To more clearly and visually present information such as the characteristics of all included studies and extracted data, the investigators summarized the characteristics of the complete included studies as well as the relevant data in Table 1.

**Table 1.** The characteristics and extracted data of all the included studies in the systematic review and meta-analysis.

Authors	Publication years	Study type	Districts	Populations	Following-up time	Adjusting factors	Treatment regimen	PPI use periods	ECOG-PS	Subgroups
Ça˘glayan et al^28^	2023	Retrospective	Turkey	Total: 86 PPI users: 45	Median 10.68 months	Age BMI Ki-67	Palbociclib + fulvestrant/aromatase inhibitors	>1/2 of the treatment	Unknown	CDKI categories Treatment lines Districts
Cosimo et al^29^	2023	RCT	Italy	Total: 416 PPI users: 91	Median 32 months	Age Metastasis ECOG	Palbociclib + fulvestrant/letrozole	Started together with CDKI >2/3 of the treatment	0-2	CDKI categories Districts
Criado et al^16^	2023	Retrospective	Spain	Total: 169 PPI users: 80	Unknown	ECOG	Palbociclib + fulvestrant/letrozole	Unknown	0-2	CDKI categories Treatment lines Districts
Del Re et al^13^	2021	Retrospective	Italy	Total: 112 PPI users: 56	Unknown	Age Menopausal status ECOG	Palbociclib + fulvestrant/letrozole	Started together with CDKI >2/3 of the treatment	0-2	Treatment lines Districts
Del Re et al^26^	2022	Retrospective	Italy	Total: 128 PPI users: 50	Unknown	Age Menopausal status Metastasis ECOG	Ribociclib + fulvestrant/letrozole	Started together with CDKI >2/3 of the treatment	0-2	Treatment lines Districts
Eser et al^27^	2022	Retrospective	Turkey	Total: 217 PPI users: 130	Unknown	Age Menopausal status Metastasis ECOG	Palbociclib/Ribociclib + fulvestrant/letrozole	>1/2 of the treatment	0-2	CDKI categories Districts
Lee et al^30^	2023	Retrospective	Korea	Total: 1310 PPI users: 344	Unknown	Age Menopausal status Prior therapy	Palbociclib + fulvestrant/letrozole	Follow-up started from PPI use >1/3 of the treatment	Unknown	CDKI categories Districts
Odabas et al^31^	2023	Retrospective	Turkey	Total: 233 PPI users: 96	Palbociclib: Median 14.1 months Ribociclib: Median 11.9 months	Age Menopausal status Metastasis ECOG	Palbociclib/Ribociclib + fulvestrant/letrozole	>2/3 of the treatment	0-3	CDKI categories Districts
Schieber et al^32^	2023	Retrospective	US	Total: 82 PPI users: 32	Unknown	Age Menopausal status ER/PR levels Metastasis ECOG	Palbociclib + fulvestrant/letrozole/Tamoxifen	Started together with CDKI >1/2 of the treatment	0-3	CDKI categories Treatment lines Districts
Takahashi et al^17^	2024	Retrospective	Japan	Total: 112 PPI users: 56	Median 31.2 months	Age Menopausal status Metastasis ECOG	Palbociclib/Abemaciclib + endocrine therapy	Started together with CDKI >1/2 of the treatment	0-2	CDKI categories Districts

Abbreviations: CDKI, cyclin-dependent kinase inhibitor; ECOG-PS, Eastern Cooperative Oncology Group Performance Status; PPI, proton pump inhibitor.

### Quality of the included studies

Prior to conducting further meta-analysis, we assessed both the quality of the study and the potential risk of bias to ensure more precise quantitative analysis outcomes. For studies assessed as low quality or with a high risk of bias, and in cases where pertinent concerns cannot be resolved, we excluded them from future meta-analyses to prevent any adverse effects on the overall study outcomes.

Two distinct methodologies were used to evaluate the quality of the studies. We assessed and ranked the quality of the studies based on the Newcastle-Ottawa Scoring Scale (Supplementary Table S1). Additionally, the quality of the data was further scrutinized through visualization using a standardized risk of bias tool (Supplementary Table S2). In total, 8 studies were classified as high-quality studies, with NOS scores ranging from 7 to 9,^13,17,26,28-32^ while 2 studies received scores between 4 and 6.^16,27^ The risk of bias evaluation produced consistent findings, with 8 studies identified as high quality also being rated as low or medium risk, whereas the remaining 2 studies were deemed to have a serious risk of bias. Notably, in the study by Eser et al,^27^ the commencement time of follow up or the initiation time of CDKI with PPI was not specified, potentially introducing an immortal time bias. Furthermore, the ECOG ratio among PPI users and non-PPI users was not provided. The study conducted by Criado et al^16^ was found to exhibit significant abnormalities, potentially introducing a confounding bias due to issues in the study design. The incomplete publication content of the study has resulted in the unavailability of crucial information regarding the study design and key outcome indicators. This absence may lead to substantial biases such as confusion bias and immortal time bias. Consequently, Eser et al’s study will be excluded from our subsequent meta-analysis concerning survival outcomes and side effects, along with Criado et al’s study data.

### Survival outcomes for concomitant PPIs and CDKIs use

**Figure 2.**
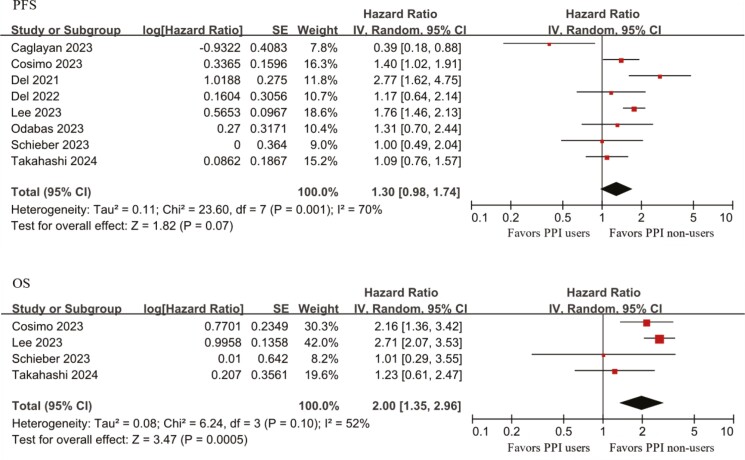
Forest plot of survival outcomes for concomitant PPIs and CDKIs use. Abbreviation: HR, hazard ratio.

Of the studies that passed the bias test, a total of 8 studies met the inclusion criteria for this group, and relevant data could be extracted.^13,17,26,28-32^ Eight studies reported the effect of concomitant PPI use on PFS when CDKIs were used concurrently in patients with breast cancer,^13,17,26,28-32^ and 4 studies reported the effect of concomitant PPI use on OS when CDKIs were used concurrently in patients with breast cancer.^17,29,30,32^ Detailed analysis results of hazard ratio for each study and pooled hazard ratio for all studies are presented in Figure 2. Our meta-analysis showed that concurrent use of PPIs was associated with a significantly shorter OS and a significantly higher risk of all-cause mortality (HR = 2.00; 95% CI, 1.35-2.96). The heterogeneity test showed that there was heterogeneity between studies (*I*² = 52%; *P* = .10). However, there was no significant association between concurrent use of PPIs and PFS and did not result in a significant change in the risk of disease progression (HR = 1.30; 95% CI, 0.98-1.74). The heterogeneity test revealed significant heterogeneity between studies (*I*² = 70%; *P* = .001).

### Survival outcomes for concomitant PPIs and CDKIs use related to specific factors

#### PFS for concomitant PPI and specific CDKI medications

In this section, we analyzed the risk of disease progression in patients with breast cancer receiving treatment with palbociclib and ribociclib while simultaneously using PPIs, based on the different types of CDKIs. The detailed analysis results of hazard ratios for each study and the summary hazard ratio for all studies are presented in Supplementary Figure S1.

Seven studies reported the impact of PPIs on the PFS of patients with breast cancer taking palbociclib.^13,20,31-35^ The results of the meta-analysis indicate that the simultaneous use of PPIs does not significantly affect the PFS of patients. There is also no significant alteration in the risk of disease progression for patients (HR = 1.40; 95% CI, 1.07-1.84). Heterogeneity testing indicates that there is heterogeneity among the studies (*I*² = 63%; *P* = .01).

Three studies reported the impact of PPIs on the PFS of patients with breast cancer taking ribociclib.^26,28,31^ The simultaneous use of PPIs did not result in a significant alteration in patients’ PFS, and the risk of disease progression did not change significantly (HR = 1.25; 95% CI, 0.80-1.95). Heterogeneity testing revealed no heterogeneity among studies (*I*² = 0%; *P* = 0.54). The study by Del et al accounted for 55.1% of the total.

#### PFS for concomitant PPIs and CDKIs use related to districts

In the 8 studies included in the meta-analysis, 2 studies were conducted in the East Asia region,^17,30^ and 6 studies were conducted in Europe or North America.^13,26,28,29,31,32^ Our meta-analysis results show that for patients with breast cancer in East Asia, the use of PPIs and CDKIs does not lead to a significant change in patient PFS (HR = 1.42; 95% CI, 0.89-2.27). Heterogeneity testing indicates heterogeneity among studies (*I*² = 81%; *P* = 0.02). Similarly, for patients with breast cancer in Europe and North America, the simultaneous use of PPIs and CDKIs also does not result in a significant change in patient progression-free survival (HR = 1.22; 95% CI, 0.80-1.87). Heterogeneity testing shows significant heterogeneity among studies (*I*² = 70%; *P* = .005) (Supplementary Figure S2).

#### PFS for concomitant PPIs and CDKIs use related to treatment lines

Among the 8 studies encompassed in the meta-analysis, 4 studies delineated the utilization of CDKIs as either first-line or second-line treatment options, followed by subsequent therapies. In 2 studies, CDKIs were used as primary treatment choices,^13,32^ whereas in another 2 studies, CDKIs were used as secondary or subsequent treatment alternatives.^28,29^ The findings of our meta-analysis indicate that among patients with breast cancer treated with CDKIs as initial therapy, the concurrent administration of PPIs does not lead to a significant change in patients PFS (HR = 1.71; 95% CI, 0.63-4.64). Heterogeneity testing revealed significant heterogeneity among the studies (*I*² = 80%; *P* = 0.03). Also, in patients with breast cancer who are prescribed CDKIs as second-line and subsequent treatment options, the concurrent administration of PPIs does not have a significant impact on the risk of disease progression. There is no statistically significant alteration in PFS for patients (HR = 0.93; 95% CI, 1.07-5.34). Heterogeneity testing revealed significant heterogeneity among the studies (*I*² = 90%; *P* = .001) (Supplementary Figure S3).

### Side effects in patients with breast cancer on PPIs and CDKIs

**Figure 3.**
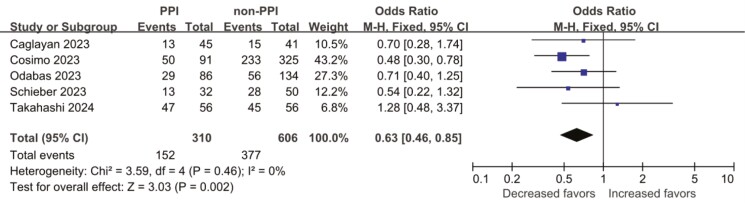
Forest plot of side effects in breast cancer patients on PPIs and CDKIs. Abbreviation: OR, odds ratio.

To examine the potential impact of concurrent PPIs on the side effects of CDKIs treatment in patients with breast cancer, we incorporated information regarding the incidence of grades 3/4 adverse events post-treatment. Five studies provided information on adverse effects,^17,28,29,31,32^ indicating that patients with breast cancer undergoing CDKIs treatment had a lower risk of developing grades 3/4 adverse events when concurrently using PPIs, with a significant reduction observed (OR = 0.63, 95% CI, 0.46-0.85). Heterogeneity testing indicated the absence of heterogeneity among the studies (*I*² = 0%; *P* = .46). The research conducted by Cosimo et al contributed to 43.2% of the total, as illustrated in Figure 3.

## Discussion

This study is the first systematic review and meta-analysis to thoroughly examine the effectiveness, adverse effects, and subgroup efficacy of simultaneously administering PPIs to patients with breast cancer undergoing CDKIs treatment. This is the first systematic review to primarily confirm the basic safety of concurrent PPI treatment in patients with breast cancer receiving CDKIs. This meta-analysis found that concurrent use of PPIs with CDKIs treatment in patients with breast cancer led to a decrease in overall survival. However, there was no statistically significant change in the crucial parameter of PFS, indicating that the use of PPIs did not affect the progression of breast cancer. When specific medications like palbociclib and ribociclib were examined individually, along with treatment lines and patient subcategories such as region or ethnicity, there was no significant change in the risk of breast cancer progression. After studying the negative effects of CDKIs treatment, it was found that giving PPIs at the same time reduced the occurrence of these side effects. The study findings can help alleviate concerns of patients and healthcare providers regarding the establishment of suitable diagnostic and treatment guidelines. This research can be a valuable resource for designing treatment plans for individuals with HR+/HER2 breast cancer, ultimately improving survival rates and the quality of life for patients.

This study differentiates itself from previous systematic reviews and meta-analyses by incorporating recent and relevant clinical findings and enhancing methodological approaches.^14,15^ It also investigates the potential effects of concurrent PPIs on specific subgroups, such as region, ethnicity, treatment regimens, among others, offering a basis for more individualized and precise medical interventions for relevant patient populations. Our results validate the fundamental safety and potential benefits of simultaneous PPIs treatment in patients with breast cancer using CDKIs, which contrasts with earlier systematic reviews. In comparison to the study by Chang et al, our research integrates the latest 3 clinical trials through a comprehensive survey, leading to variations in the outcomes related to palbociclib in combination with PPIs. Additionally, we conducted an analysis of CDKIs as a whole and a more detailed subgroup analysis. In contrast to de Moraes et al’s review, our survival outcomes for CDKIs used concurrently with PPIs and the outcomes for specific medications exhibit significant disparities. While acknowledging the valuable contributions of de Moraes et al’s review at the time, it is noted that they did not include all relevant clinical studies, such as the study by Takahashi et al^17^ Besides, in terms of quality assessment, we used a novel GRADE evaluation method in addition to the NOS scale for scoring, identifying a serious risk of bias in the studies by Eser et al^27^ and Criado et al,^16^ which were consequently excluded from the subsequent analysis to prevent potential bias influencing the results. We contend that our study adheres to the latest systematic review and meta-analysis standards and methodologies, yielding conclusions with a higher level of evidence and persuasiveness. These findings are expected to offer clinicians a fresh reference and guidance when devising treatment strategies for HR+/Her2− patients with breast cancer.

As previously indicated, CDKIs, which are orally administered as weakly basic medications in tablet or capsule formulations, may experience altered absorption due to changes in gastric pH and the gastrointestinal microenvironment resulting from the concomitant use of PPIs.^10,11,13^ Nevertheless, it is crucial to acknowledge that an analysis of the effects of PPIs on CDKIs solely based on drug absorption in the gastrointestinal tract is inherently incomplete. It is the bioavailability of a drug that stands as one of the crucial indicators to be considered when assessing its impact within the body. Several preclinical and clinical studies have examined the bioavailability of CDKIs in conjunction with PPIs. Samant et al^33^ reported that PPIs have a negligible effect on the maximum plasma concentration (*C*_max_) and area under the curve (AUC) values of ribociclib, indicating a minimal impact on its bioavailability. In the study by Sun et al, it was indicated that the co-administration of rabeprazole with palbociclib resulted in a decrease of 62% and 41% in *C*_max_ values and 80% and 13% in AUC values in the fasting or fed states, respectively.^34^ Nevertheless, Yu et al demonstrated that the AUC and *C*_max_ of palbociclib, when co-administered with PPIs, remain within the bioequivalence thresholds, which indicated that the bioavailability of palbociclib remained unaffected.^35^ Furthermore, PPIs act as inhibitors of the efflux transporter P-glycoprotein (P-gp) for CDKIs, potentially leading to elevated CDKI concentrations within cells through efflux inhibition, as evidenced by in vitro studies.^36,37^ Based on the findings of these experiments and clinical evidence, it is our contention that while PPIs may have some impact on the absorption of CDKIs, the concentration of the drug at the specific site of CDKI action is unlikely to be substantially altered. Consequently, the survival outcomes of patients did not experience significant alterations.

The heightened risk of all-cause mortality in patients with breast cancer undergoing CDKI treatment and using PPIs is likely associated with the patients’ individual health conditions. It is widely recognized that variables such as the patient’s physical health and the prognosis of cancer can exert unpredictable yet substantial influences on the outcomes of pharmacological treatment.^38,39^ None of the clinical studies included in this analysis fully reported or matched patients with cancer. Also, in these retrospective studies, cancer patients treated with PPIs typically exhibit poorer physical conditions. Hence, the variations in OS could potentially stem from variances in the patients’ individual cancer types and physical health conditions rather than being solely attributed to the concurrent use of PPIs. This necessitates the acquisition of more comprehensive data or the conduct of additional analysis via randomized controlled trials (RCTs).

It is found that the HR for PFS of palbociclib was higher compared to ribociclib. This difference may be attributed to the ratio of free mean steady-state concentration (*C*_ss_) to in vitro cellular potency (*C*_ss_/IC50), which was 0.94 for palbociclib and over 25^33,40^ for ribociclib. This indicates that ribociclib’s therapeutic site concentration is less influenced by drug absorption than palbociclib, resulting in its efficacy being less impacted by the co-administration of proton pump inhibitors (PPIs). An additional noteworthy finding is the variability within palbociclib itself. The currently approved palbociclib is available in both capsule and tablet forms. Capsules were used earlier, while tablets did not receive FDA approval for clinical use until 2020.^32^ Variations in absorption kinetics between the 2 forms have raised concerns among researchers regarding potential differences in the effects of different palbociclib formulations when combined with PPIs in patients with breast cancer. Among the studies analyzed, 5 examined the impact of PPIs on PFS in patients with breast cancer using palbociclib capsules. The results indicated that the use of PPIs did not significantly alter patients’ PFS (HR = 1.49; 95% CI, 1.12-1.99).^13,17,29-31^ Two studies investigated the effect of PPIs on PFS in patients with breast cancer taking palbociclib tablets, also with no significant change observed in PFS (HR = 0.86; 95% CI, 0.47-1.57) (Supplementary Figure S4).^17,32^ Although the overall outcomes were unaffected, it was evident that the HR of PFS was higher in patients using palbociclib capsules compared to tablets. This suggests that the observed impact of PPI co-administration on palbociclib efficacy in patients with breast cancer may be influenced by absorption kinetics related to the drug formulation, rather than the bioavailability of the palbociclib molecule itself in the presence of PPIs. These findings offer insights for clinical decision-making regarding palbociclib, indicating that when PPIs are necessary for patients with breast cancer, the use of palbociclib tablets may be a preferable option. Abemaciclib, another type of CDKIs, has been less studied in combination with PPIs in patients with breast cancer due to its recent introduction as a new drug. Only Takahashi et al have investigated the outcomes of this combination, revealing no increased risk of death or disease progression in patients with breast cancer (PFS: HR = 1.30; 95% CI, 0.53-3.17 and OS: HR = 1.22; 95% CI, 0.33-4.47).^17^ Further clinical research on Abemaciclib is warranted.

Common adverse effects associated with CDKIs encompass hematologic manifestations and gastrointestinal symptoms such as diarrhea. The side effects are categorized into levels 1-4 according to their severity, with 3/4-level side effects presenting a higher risk to patients, thus garnering considerable interest from researchers.^4^ According to the preceding analysis, the concurrent administration of PPIs may not result in substantial alterations in the systemic levels of CDKIs, indicating that the change in occurrence of side effects may not induce by CDKIs per se. It is probably PPIs that exhibit a protective influence on certain side effects induced by CDKIs, particularly gastrointestinal side effects. Hence, the concurrent use of PPIs with CDKIs for treatment substantially reduces the incidence of 3/4-level side effects in patients.

While this study made efforts to analyze all clinical research data in accordance with regulatory requirements, it is important to acknowledge certain limitations. The main clinical studies in this investigation were retrospective cohort studies, mainly sourced from databases and questionnaires, which could introduce recall bias and information bias. In addition to study design, the absence of essential information and the presence of confounding variables significantly affect research results. Despite efforts to include all relevant clinical research on the subject, the number of related studies is limited due to CDKIs being a new treatment approach, especially for specific subgroups. This situation may lead to potential publication bias or bias from small-scale studies. Finally, it is important to acknowledge the presence of significant heterogeneity in certain study outcomes. The variability may stem from variations in the comprehensive treatment protocols administered by diverse institutions and patients, as well as variances in the physical health and cancer profiles of the patients involved.

## Conclusion

This study revealed that the concurrent use of PPIs in patients with breast cancer receiving CDKIs may increase the risk of all-cause mortality. However, the more crucial parameter, the risk of disease progression in patients, does not exhibit statistically significant alterations, which indicates the basic safety of the relevant treatment plans. Moreover, the concurrent administration of PPIs can significantly reduce the adverse effects associated with CDKIs therapy. This information has the potential to alleviate the apprehensions of healthcare providers in devising treatment strategies and to reassure patients. Nonetheless, given the limitations of the preceding discussion, further high-quality clinical studies on this subject are still warranted.

## Supplementary Material

oyae320_suppl_Supplementary_Tables_1-2_Figures_1-4

## Data Availability

Data sharing is not applicable to this article as no datasets were generated or analyzed during the current study.
