# Supplementary material for: Efficacy of cyclin-dependent kinase inhibitors with concurrent proton pump inhibitors in patients with breast cancer: a systematic review and meta-analysis
Source: Oncologist. 2025 Feb 18;30(2):oyae320. doi: 10.1093/oncolo/oyae320 (PMC11833246; doi:10.1093/oncolo/oyae320)

**Supplementary Table 1: The result of Newcastle–Ottawa quality assessment**

| Studies | Selection | | | | Comparability | Exposure | | | Scores |
| --- | --- | --- | --- | --- | --- | --- | --- | --- | --- |
|  | Adequente definition of cases | Representativeness of the cases | Selection of controls | Definition of controls | Control of important factor | Ascertainment of exposure | Same method of ascertainment for cases and controls | Non-responserate |  |
| Caglayan 2023 | ★ | ★ | ☆ | ★ | ★★ | ★ | ★ | ★ | 8 |
| Cosimo 2023 | ★ | ★ | ★ | ★ | ★★ | ★ | ★ | ★ | 9 |
| Criado 2023 | NA | NA | NA | NA | NA | NA | NA | NA | 0 |
| Del 2021 | ★ | ★ | ★ | ★ | ★☆ | ★ | ★ | ★ | 8 |
| Del 2022 | ★ | ★ | ★ | ★ | ★☆ | ★ | ★ | ★ | 8 |
| Eser 2022 | ★ | ☆ | ☆ | ★ | ★☆ | ★ | ★ | ★ | 6 |
| Lee 2023 | ★ | ★ | ★ | ★ | ★★ | ★ | ★ | ★ | 9 |
| Odabas 2023 | ★ | ★ | ★ | ★ | ★★ | ★ | ★ | ★ | 9 |
| Schieber 2023 | ★ | ★ | ★ | ★ | ★☆ | ★ | ★ | ★ | 8 |
| Takahashi 2024 | ★ | ★ | ★ | ★ | ★☆ | ★ | ★ | ★ | 8 |

★ = 1 scores; ☆ = 0 scores

**Supplementary Table 2: The standardized bias tool**

| Study ID | Bias due to confunding | Bias due to selection of participants | Bias in classification of interventions | Bias due to deviations from intended interventions | Bias due to missing data | Bias in measurement of outcomes | Bias in selection of the reported result | Overall | Weight |
| --- | --- | --- | --- | --- | --- | --- | --- | --- | --- |
| Caglayan 2023 | Medium | Low | High | High | High | High | High | Low | 86 |
| Cosimo 2023 | High | High | High | High | High | High | High | High | 486 |
| Criado 2023 | / | / | / | / | / | / | / | / | NA |
| Del 2021 | Medium | High | High | High | High | High | High | Medium | 112 |
| Del 2022 | Medium | High | High | High | High | High | High | Medium | 128 |
| Eser 2022 | High | Low | Low | Low | Low | Low | Low | Low | 217 |
| Lee 2023 | High | High | High | High | High | High | High | High | 344 |
| Odabas 2023 | Medium | Medium | High | High | High | High | High | Medium | 220 |
| Schieber 2023 | Medium | High | High | High | High | High | High | Medium | 88 |
| Takahashi 2024 | High | Low | Low | Low | Low | Low | Low | Low | 240 |

The code is as follows:

library("robvis")

library(ggplot2)

setwd(dir="/Users/zhuziyu/")

data <- read.csv("/Users/zhuziyu/rob1.csv")

data

#ROB2,ROBINS-I,QUADAS-2

rob_summary(data = data,tool="ROB1",overall = TRUE,weighted = TRUE,colour=c("orange","green","skyblue"))

rob_summary(data = data,tool="ROB1",overall = TRUE,weighted = FALSE,colour=c("orange","green","skyblue"))+ggtitle("??????","??????")+theme(plot.title = element_text(hjust=0.5))

rob_traffic_light(data = data,tool="ROB1",colour=c("orange","green","skyblue"),psize = 10)+theme(plot.title = element_text(hjust=0.5))

rob_summary(data = data,tool="ROB1",overall = TRUE,weighted = FALSE,colour=c("orange","green","skyblue"))+ggtitle("??????","??????")+theme(plot.title = element_text(hjust=0.5))

rob_traffic_light(data = data,tool="ROB1",colour=c("orange","green","skyblue"),psize = 10)+ggtitle("??????","??????")+theme(plot.title = element_text(hjust=0.5))

**Supplementary Figure 1: Forest plot of PFS for concomitant PPI and specific CDKI medications**


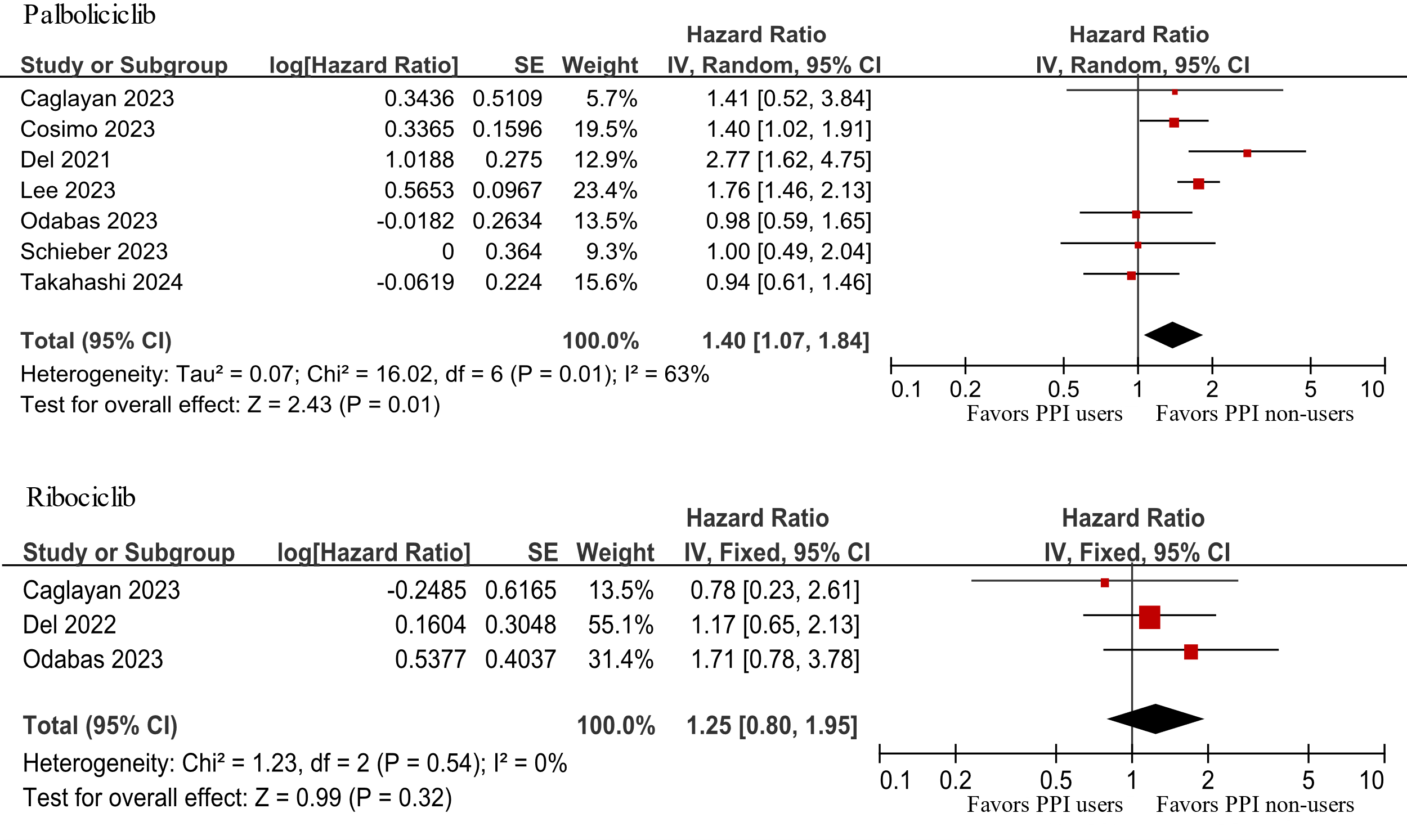


**Supplementary Figure 2: Forest plot of PFS for concomitant PPIs and CDKIs use related to districts**


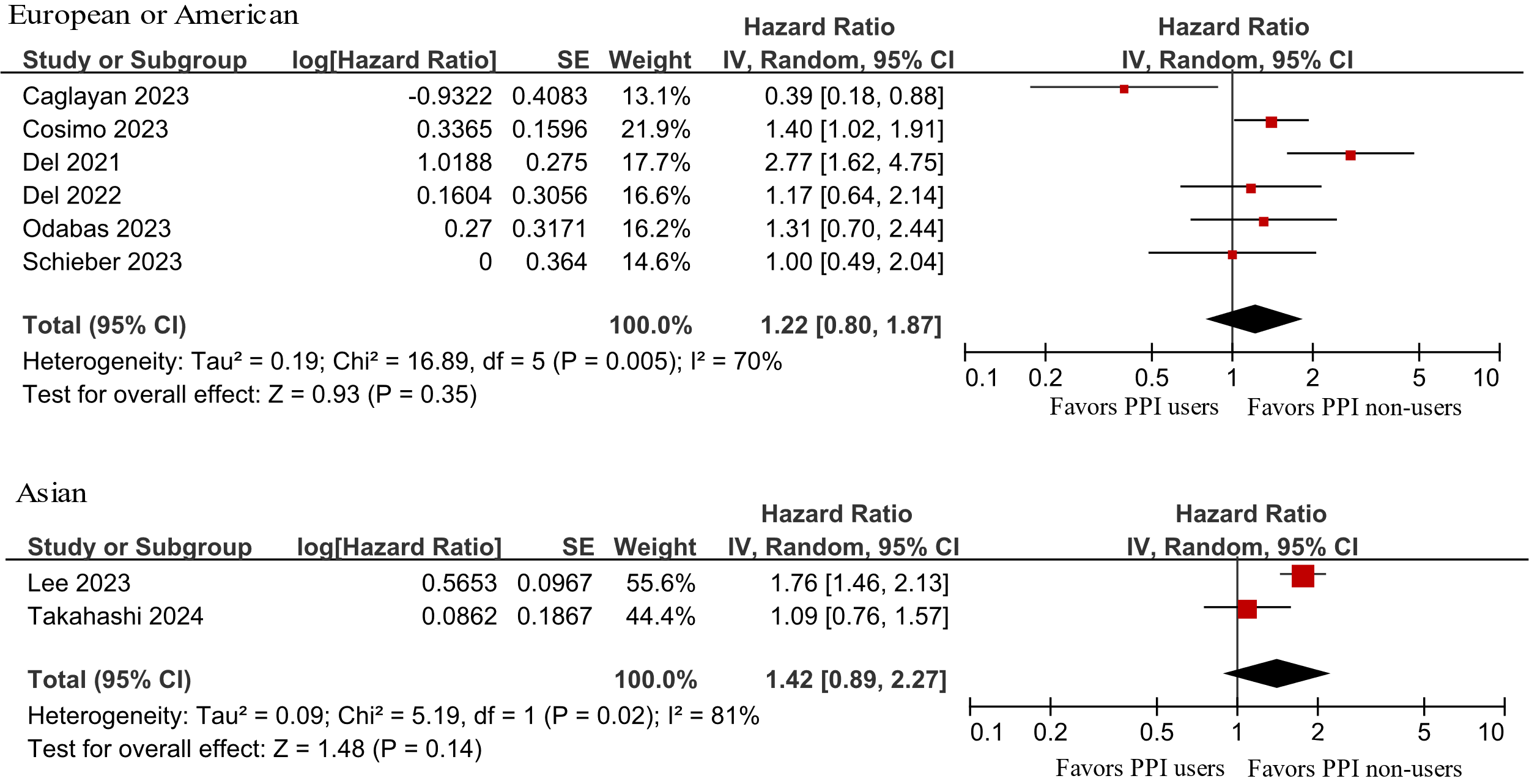


**Supplementary Figure 3: Forest plot of PFS for concomitant PPIs and CDKIs use related to treatment lines**


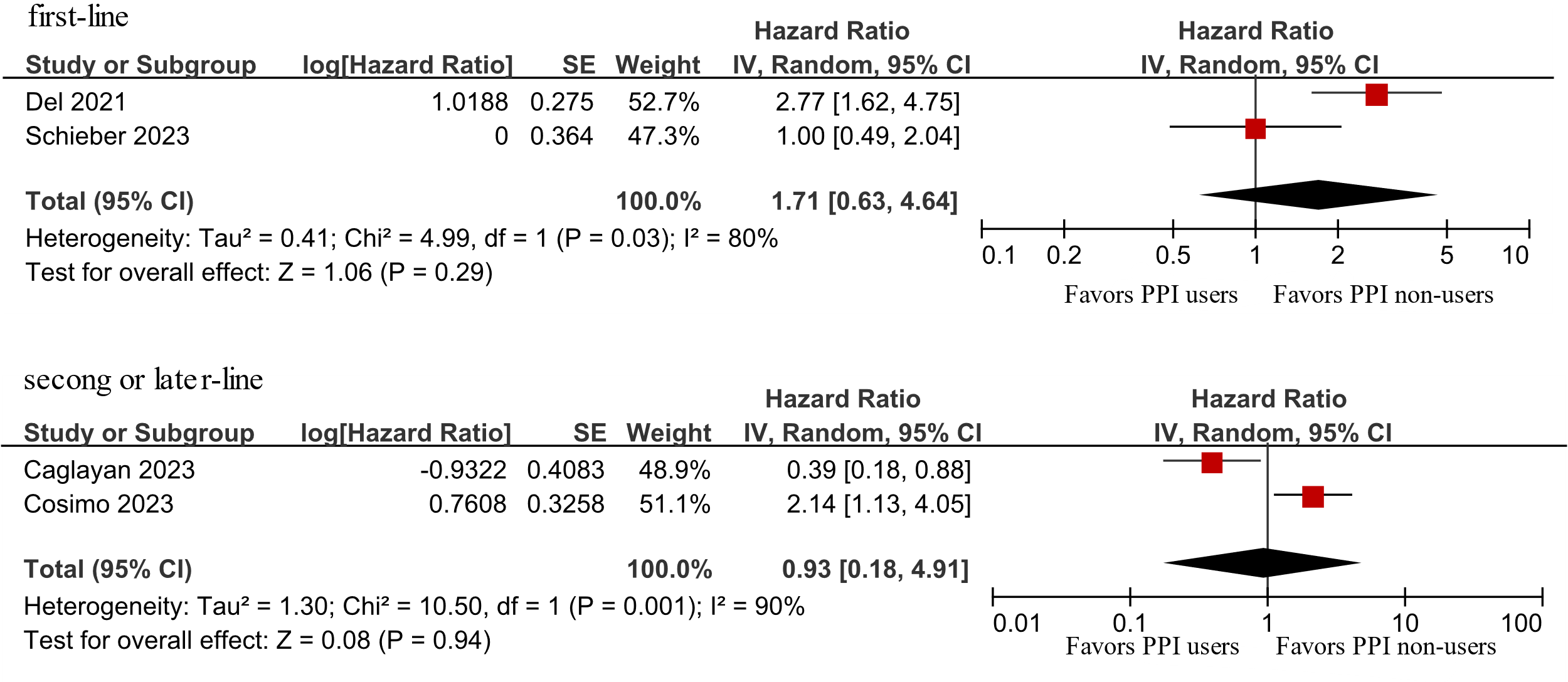


**Supplementary Figure 4: Forest plot of PFS for concomitant PPIs and different Palbociclib forms**


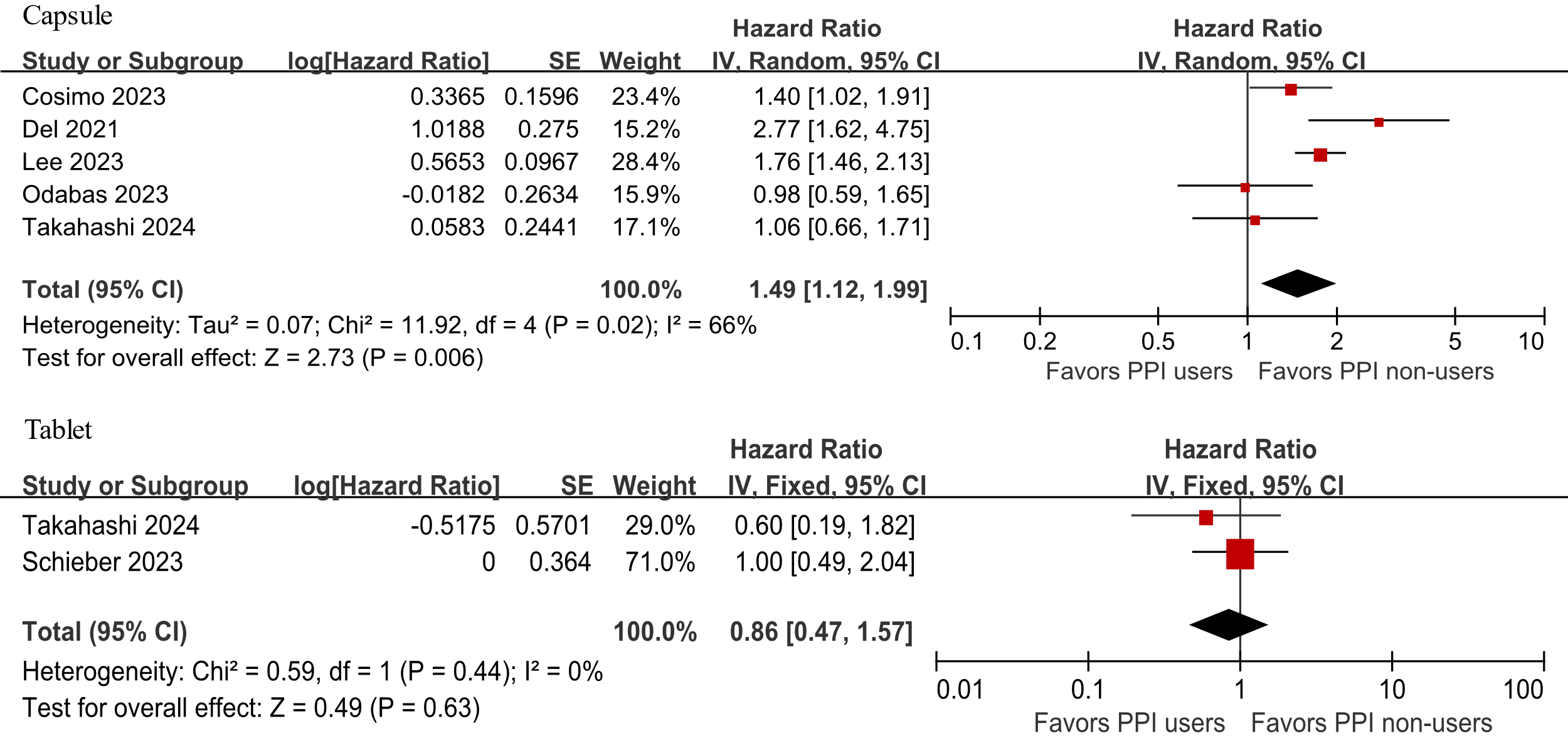

Supplement: oyae320_suppl_Supplementary_Tables_1-2_Figures_1-4 [file oyae320_suppl_supplementary_tables_1-2_figures_1-4.docx]
